# Combined effect of inter-arm systolic blood pressure difference and carotid artery plaque on cardiovascular diseases and mortality: A prospective cohort study

**DOI:** 10.3389/fcvm.2022.904685

**Published:** 2022-11-10

**Authors:** Cuijuan Yun, Qian Xin, Sijin Zhang, Shuohua Chen, Jianli Wang, Chi Wang, Miao Wang, Maoxiang Zhao, Yizhen Sun, Ziwei Hou, Shouling Wu, Hao Xue

**Affiliations:** ^1^School of Medicine, Nankai University, Tianjin, China; ^2^Department of Cardiology, The Sixth Medical Center, Chinese People’s Liberation Army General Hospital, Beijing, China; ^3^Department of Cardiology, Kailuan General Hospital, Tangshan, China

**Keywords:** inter-arm systolic blood pressure difference, carotid artery plaque, cardiovascular disease, all-cause mortality, combined effect

## Abstract

**Objectives:**

Previous studies have confirmed the relations between inter-arm systolic blood pressure difference (IASBPD) and carotid artery plaque with the risk of cardiovascular diseases (CVD). But it is unclear whether the combined effect of IASBPD and carotid artery plaque further increases the risk of CVD and all-cause mortality.

**Materials and methods:**

We enrolled 4,970 participants (≥40 years old) in the prospective Kailuan study. All participants underwent dual-arm blood pressure and carotid artery ultrasounds. IASBPD was the absolute value of the difference between dual-arm blood pressure. All the participants were divided into four groups according to their IASBPD levels and the presence or absence of carotid artery plaque and Cox proportional hazards models were used to calculate the hazard ratios (HRs) and 95% confidence interval (CI) for incident CVD and all-cause mortality.

**Results:**

During a median follow-up of 7 years, 179 CVD events and 266 deaths occurred. Multivariable Cox Regression showed that participants with IASBPD ≥ 10 mmHg and plaque had a significantly higher incidence of CVD, cerebral infarction (CI), and myocardial infarction (10, 7.27, and 1.36%, respectively). After adjusting for covariates, the IASBPD ≥ 10 mmHg and carotid plaque group significantly increased risks for CVD (HR 2.38; 95% CI, 1.40∼4.05), CI (HR, 2.47; 95% CI, 1.31∼4.67), and all-cause mortality (HR, 2.08; 95% CI, 1.20∼3.59).

**Conclusion:**

Our study indicated that the combination of IASBPD and carotid artery plaque was associated with incident CVD and all-cause mortality.

## Introduction

Cardiovascular disease (CVD) is the leading cause of death in China and has become an important public health problem. Atherosclerosis is the main cause of coronary heart disease, cerebral infarction (CI), and peripheral vascular disease (1), and is also the pathological basis of cardiovascular and cerebrovascular events development. People with multiple vascular atherosclerosis had a significantly increased risk of CVD compared with those with single atherosclerosis. Therefore, in order to improve the accuracy of CVD risk prediction, researchers tried to integrate different vascular markers into traditional risk prediction models.

Carotid artery plaque is a marker of degenerative changes and structural changes in atherosclerosis. The previous study shows that the presence of carotid plaque is associated with the risk of stroke, myocardial infarction (MI), and mortality (2, 3). Meta-analysis shows that carotid plaque is also a better predictor of future cardiovascular events than the intima-media thickness (4). In addition, a multi-ethnic study of atherosclerosis in the United States shows that carotid plaque is associated with future stroke or transient ischemic attack (5). Inter-arm Systolic Blood Pressure Difference (IASBPD) means the systolic blood pressure difference between the two arms. The International Hypertension Guideline recommends checking blood pressure (BP) on both arms when measuring BP and recognizes the association of systolic interarm blood pressure differences with cardiovascular risk (6). Recent studies have found that IASBPD, especially 10 mmHg or more, is associated with subclavian stenosis, peripheral arterial disease, cerebrovascular disease, CVD, and all-cause mortality (7–10).

Previous studies have confirmed the relations between IASBPD and carotid artery plaque with the risk of CVD and all-cause mortality, respectively. However, the contribution of the combination of IASBPD and carotid artery plaque to increase the risk of CVD is not clear. Therefore, in order to address this issue, we prospectively investigate the association of the combination of IASBPD and carotid artery plaque with the risk of CVD and all-cause mortality in the Kailuan study.

## Materials and methods

### Study design and participants

**FIGURE 1:**
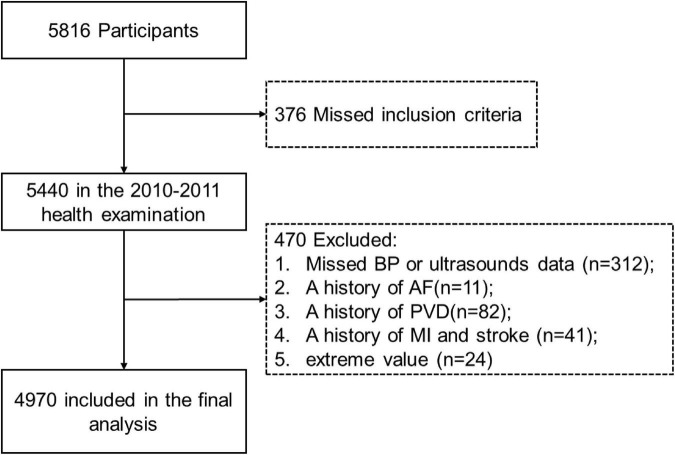
Selection of study population. BP, blood pressure; AF, atrial fibrillation; PVD, peripheral vascular disease; MI, myocardial infarction.

The study was a community-based prospective cohort (11–13). As detailed elsewhere, from June 2006 to September 2007 (visit 1) and June 2008 to September 2009 (visit 2), the employer of Tangshan Kailuan Company sponsored two times of health examinations for all 101,510 employees (81,110 men and 20,400 women, aged: 18–98 years) (14). A total of 5,816 participants were selected as an observational cohort using a random stratified sampling method and the response rate was 99%. A total of 5,440 individuals met the inclusion criteria (≥40 years old; no history of stroke, transient ischemic attack, or MI; no serious physical disability) and participated in the 2010–2011 health examination (visit 3). Finally, 4,970 adults were included in the analysis after excluding the following subjects: (a) the absence of information about brachial BP or sonography examination (*n* = 312); (b) a history of atrial fibrillation (*n* = 11); (c) a history of peripheral vascular disease (*n* = 82); (d) a history of MI and stroke (*n* = 41); (e) extreme value (*n* = 24). Data for 4,970 employees (2,960 men and 2,010 women) aged 18–96 years (55.00 ± 11.72) were used for the present analysis (a flowchart of participant inclusion appears in Figure 1). The study (ChiCTR-TNC-11001489) was performed according to the guidelines of the 1964 Declaration of Helsinki, and was approved by the Ethics Committee of the Kailuan Medical Group, Kailuan Company. Written informed consent had been given by all participants.

### Demographic and clinical data collection

The medical and family history of all participants was obtained with a questionnaire including information about demographic data and occupational status, behavior (sleep, smoking, drinking alcohol, exercise, and diet habits), past medical history, and family history. Questionnaires were administered face-to-face by investigators. Height, weight, and waist circumference were measured and body mass index was calculated as kg/m^2^. The biochemical tests included total cholesterol, triglyceride, fasting blood glucose, high-density lipoprotein, and low-density lipoprotein.

### Measurement of blood pressure and inter-arm systolic blood pressure difference

We simultaneously measured the BP in four extremities, in the supine position after at least 5 min bed rest using an arteriosclerosis detection device (Model BP203RPE III, Omron, China), for each participant detected twice and averaged for the final results. IASBPD was defined as the absolute value of the right arm minus the left arm systolic BP.

### Ultrasonic testing of carotid plaque

Carotid artery ultrasonography used a diagnostic ultrasonic instrument (HD-15, PHILIPS, China), with a high-frequency probe and frequency of 5–12 mHz. The carotid artery and bifurcation, the origin of the internal carotid artery, and the external carotid artery were routinely scanned, and the intima and media thickness were measured to observe the existence of plaque, and the location and size of plaque were recorded.

Carotid artery plaque was defined as invading the arterial lumen by at least 0.5 mm or 50% of the surrounding intima–media thickness value or showing a thickness > 1.5 mm from the media-adventitia interface to the intima–lumen interface (15). The ultrasound images were jointly judged by two ultrasound doctors with more than 5 years of working experience according to the above criteria and were randomly rechecked by different ultrasound doctors to judge intima-media thickness and plaque again (retest reliability was 97%).

### Definitions of other cardiovascular disease risk factors

Cardiovascular disease events, including MI and CI, were ascertained by investigating the annual discharge form from local hospitals and death certificates of state vital statistics offices and by contacting participants annually for a history of CVD events, in the Kailuan study. MI was diagnosed based on the patient’s clinical symptoms, electrocardiograph changes, and dynamic myocardial enzyme changes in line with the World Health Organization’s Multinational Monitoring of Trends and Determinants in Cardiovascular Disease Criteria (16). For patients with CI, the diagnosis depends on the signs and symptoms and photocopied neuroimages (computed tomography or magnetic resonance imaging) and other diagnostic reports, in line with the World Health Organization criteria (17). According to the American Diabetes Association (18), the diagnostic criteria for Diabetes were defined as fasting blood glucose ≥ 7.0 mmol/L, random blood glucose ≥ 11.1 mmol/L, or taking hypoglycemic drugs. Hypertension was diagnosed as follows: systolic BP ≥ 140 mmHg and/or diastolic BP ≥ 90 mmHg or the use of an antihypertensive medication following the JNC7 Guideline (19). All-cause death data were obtained from the Kailuan Social Security Information system every year.

### Statistical analyses

Statistical analyses were performed using SAS version 9.4 (SAS Institute). According to IASBPD and carotid plaque, the participants’ objects were divided into four groups: IASBPD < 10 mmHg without carotid plaque; IASBPD ≥ 10 mmHg without carotid plaque; IASBPD < 10 mmHg with carotid plaque; and IASBPD ≥ 10 mmHg with carotid plaque. Continuous variables were described as means ± standard deviations and were compared by analysis of variance (ANOVA). Categorical variables were exhibited as frequencies and percentages and were compared using the chi-square test. Multivariate Cox proportional hazards regression was used to estimate the risks of cardiovascular events. The incidence of events was estimated using the Kaplan-Meier method. A *P*-value of less than 0.05 was considered statistically significant.

## Results

### Baseline characteristics of the study population

The baseline characteristics of the study population are shown in Table 1. In this study, 4,790 participants (mean age 55.00 ± 11.72 years, 2,960 males) were concluded. The proportion of smoking, drinking alcohol, hypertension, and Hyperlipidemia and the average value of blood pressure and fasting blood glucose were higher in the group of IASBPD ≥ 10mmHg with plaque, compared with other groups.

**TABLE 1:** Baseline characteristics of the study population.

	IASBPD < 10 mmHg without plaque (*n* = 2,623)	IASBPD ≥ 10 mmHg without plaque (n = 244)	IASBPD < 10 mmHg with plaque (*n* = 1,883)	IASBPD ≥ 10 mmHg with plaque (n = 220)	*P*
Age (years)	50.08 ± 8.16	48.43 ± 7.30	62.25 ± 12.35	58.66 ± 11.73	<0.01
Male (*n*, %)	1,231 (46.93%)	164 (67.21%)	1,394 (74.03%)	171 (77.73%)	<0.01
BMI (kg/m^2^)	24.73 ± 3.17	26.67 ± 3.70	24.73 ± 3.15	26.58 ± 3.29	<0.01
Rt. arm SBP (mmHg)	128.76 ± 16.92	138.29 ± 19.18	143.05 ± 20.03	150.38 ± 23.55	<0.01
Lt. arm SBP (mmHg)	77.97 ± 11.23	83.18 ± 12.19	83.21 ± 11.50	87.00 ± 12.80	<0.01
Rt. arm DBP (mmHg)	128.17 ± 17.04	143.22 ± 20.36	142.66 ± 20.08	153.24 ± 23.66	<0.01
Lt. arm DBP (mmHg)	77.55 ± 11.27	83.18 ± 13.24	82.81 ± 11.49	86.81 ± 13.33	<0.01
FBG (mmol/L)	5.40 ± 1.27	5.54 ± 1.30	5.80 ± 1.71	5.86 ± 1.93	<0.01
LDL-C (mmol/L)	2.55 ± 0.74	2.74 ± 0.70	2.71 ± 0.81	2.68 ± 0.75	<0.01
Smoking (*n*, %)	801 (30.54%)	96 (39.34%)	845 (44.88%)	112 (50.91%)	<0.01
Drinking alcohol (*n*, %)	343 (13.08%)	47 (19.26%)	275 (14.6%)	48 (21.82%)	<0.01
Diabetes (*n*, %)	191 (7.28%)	20 (8.2%)	335 (17.79%)	39 (17.73%)	<0.01
Hypertension (*n*, %)	805 (30.69%)	124 (50.82%)	1,177 (62.51%)	166 (75.45%)	<0.01
Hyperlipidemia (*n*, %)	246 (9.38%)	19 (7.79%)	248 (13.17%)	30 (13.64%)	<0.01
Antihypertensive treatment (*n*, %)	300 (11.44%)	27 (11.07%)	554 (29.42%)	63 (28.64%)	<0.01

IASBPD, inter-arm systolic blood pressure difference; BMI, body mass index; SBP, systolic blood pressure; DBP, diastolic blood pressure; FBG, fasting blood glucose; LDL-C, low-density lipoprotein cholesterol; Rt., right; Lt., left; *n*, number.

### Association of the combined effect of inter-arm systolic blood pressure difference and carotid artery plaque on cardiovascular diseases events and all-cause mortality

During the follow-up period of 4 years, 179 cases of CVD events (including 127 cases of CI and 42 cases of MI) and 266 deaths occurred. Compared with other groups, participants with IASBPD ≥ 10 mmHg with plaque had a significantly higher incidence of CVD, CI, and MI (10, 7.27, and 1.36%, respectively). The cumulative incidence of all-cause mortality significantly increased in the IASBPD < 10 mmHg in the carotid plaque group; however, the two groups with a plaque were higher than the groups without plaque for all-cause mortality (Table 2).

**TABLE 2:** Incidence of cardiovascular disease (CVD) events and all-cause mortality among the groups.

	IASBPD < 10 mmHg without plaque (*n* = 2,623)	IASBPD ≥ 10 mmHg without plaque (*n* = 244)	IASBPD < 10 mmHg with plaque (*n* = 1,883)	IASBPD ≥ 10 mmHg with plaque (*n* = 220)	*P*
CVD	51 (1.94%)	5 (2.05%)	101 (5.36%)	22 (10%)	<0.01
All-cause mortality	49 (1.87%)	6 (2.46%)	191 (10.14%)	20 (9.09%)	<0.01
CI	33 (1.26%)	3 (1.23%)	75 (3.98%)	16 (7.27%)	<0.01
MI	14 (0.53%)	1 (0.41%)	24 (1.27%)	3 (1.36%)	<0.05

IASBPD, inter-arm systolic blood pressure difference; CVD, cardiovascular disease; CI, cerebral infarction; MI, myocardial infarction, *n* = number.

The IASBPD < 10 mmHg without carotid plaque group was defined as a control group. We conducted the univariate regression analysis of the relevant risk factors, refer to Appendix 1 for details. Multivariable Cox Regression evaluated the association of the combined effect of IASBPD and carotid artery plaque with CVD events and all-cause mortality (Table 3). After adjusting for confounding factors (including age, gender, body mass index, smoking, drinking alcohol, hypertension, diabetes, taking hypotensive drugs, total cholesterol, and high-density lipoprotein), we did not find an increased risk in the group of IASBPD ≥ 10 mmHg without carotid plaque compared with IASBPD < 10 mmHg without carotid plaque group. The IASBPD < 10 mmHg and carotid plaque group showed the risk of all-cause mortality was 1.73 times higher (95% CI, 1.20∼2.48). The IASBPD ≥ 10 mmHg and carotid plaque group significantly increased risks for CVD (HR 2.38; 95% CI, 1.40∼4.05), CI (HR, 2.47; 95% CI, 1.31∼4.67), and all-cause mortality (HR, 2.08; 95% CI, 1.20∼3.59), but the risk for MI was no significant difference.

**TABLE 3:** Multivariable Cox regression analysis for cardiovascular disease events and all-cause mortality.

		Model 1		Model 2		Model 3	
				
		*P*	HR (95% CI)	*P*	HR (95% CI)	*P*	HR (95% CI)
CVD	1		Reference		Reference		Reference
	2	>0.05	1.04 (0.42∼2.62)	>0.05	0.90 (0.36∼2.27)	>0.05	0.78 (0.31∼1.98)
	3	<0.01	2.00 (1.36∼2.93)	<0.01	1.84 (1.25∼2.70)	<0.01*	1.43 (0.97∼2.10)
	4	<0.01	4.17 (2.48∼7.00)	<0.01	3.17 (1.87∼5.39)	<0.01*	2.38 (1.40∼4.05)
All-cause mortality	1		Reference		Reference		Reference
	2	>0.05	1.63 (0.70∼3.81)	>0.05	1.66 (0.71∼3.89)	>0.05	1.59 (0.68∼3.74)
	3	<0.01	1.86 (1.31∼2.66)	<0.01	1.86 (1.30∼2.66)	<0.01*	1.73 (1.20∼2.48)
	4	<0.01	2.32 (1.36∼3.94)	<0.01	2.25 (1.30∼3.88)	<0.01*	2.08 (1.20∼3.59)
CI	1		Reference		Reference		Reference
	2	>0.05	0.99 (0.30∼3.22)	>0.05	0.87 (0.27∼2.88)	>0.05	0.78 (0.31∼1.98)
	3	<0.01	2.19 (1.37∼3.49)	<0.01	1.99 (1.25∼3.16)	>0.05	1.50 (0.94∼2.39)
	4	<0.01	4.50 (2.42∼8.37)	<0.01	3.35 (1.78∼6.31)	<0.01*	2.47 (1.31∼4.67)
MI	1		Reference		Reference		Reference
	2	>0.05	0.71 (0.09∼5.44)	>0.05	0.60 (0.08∼4.62)	>0.05	0.50 (0.07∼3.91)
	3	<0.01	1.76 (0.83∼3.75)	>0.05	1.55 (0.73∼3.32)	>0.05	1.36 (0.63∼2.94)
	4	<0.01	1.99 (0.56∼7.11)	>0.05	1.56 (0.43∼5.65)	>0.05	1.25 (0.34∼4.58)

Model 1 corrected for age and gender. Model 2 further corrected BMI, total cholesterol (TC), and high-density lipoprotein (HDL) based on Model 1. Model 3 further corrected smoking, drinking alcohol, hypertension, diabetes, and taking hypotensive drugs based on Model 2. HR, hazard ratios; 95% CI, 95% confidence interval; CVD, cardiovascular disease; CI, cerebral ischemia; MI, myocardial infarction. *Statistically significant (*P* < 0.05).

## Discussion

The present study confirmed that carotid artery plaque was an independent risk factor for all-cause mortality in the general population. The combination of IASBPD and carotid artery plaque improved the prognostic value.

Carotid plaque is considered a surrogate marker of subclinical atherosclerosis and is associated with the risk of stroke, MI, and mortality (2, 3, 20). In the present results, we found that carotid artery plaque significantly increased the risk of all-cause mortality by 1.73 times in the general Chinese population. This finding is consistent with the results reported in a prospective cohort study based on a community of 2,956 Chinese adults, which found that persons with carotid plaque had a higher risk for mortality (21). Besides, the Atherosclerosis Risk in Communities and the Cardiovascular Health Study have demonstrated that carotid plaque was associated with a 35% increased risk of sudden cardiac death (22). Atherosclerotic plaques not only increased the risk for all-cause mortality but also increased the risk for cardiac and cerebrovascular events. The Suita Study conducted in the general Japanese population indicated that the progression of incident carotid plaque increased the risk of incident CVD and stroke by 1.95 and 2.01 times, respectively (23). Another research found that compared with people without plaque, the total risk of stroke was 1.58 and the risk of cardiovascular disease was 1.71 (3). However, the presence of carotid plaque alone in our study was marginally significant in predicting cardiovascular risk, suggesting carotid plaque alone may be limited in predicting CVD in the Chinese general population, and the reason for this result may be related to a smaller sample size of this group with carotid plaque alone and our study included more males.

International hypertension guidelines recommend checking BP on both arms for the first time, choosing the arm with higher BP to measure subsequently for the detection and management of hypertension (6, 19). As the guidelines mentioned, IASBPD is a simple, accessible risk marker for cardiovascular events in patients with hypertension (6). On the other hand, IASBPD was concerned about patients with diabetes, chronic kidney disease, coronary heart disease, peripheral vascular disease, or other populations relevant to primary care (7–9, 24). Studies further demonstrated that the increase of IASBPD was still associated with the additional risk of CVD after adjustment for the Framingham risk scores, Atherosclerotic Cardiovascular Disease, or cardiovascular risk score scores (9, 25, 26). Therefore, IASBPD may be more efficacy for the prediction of CVD events and mortality than the existing cardiovascular risk scores. Consistent with guidelines for hypertension and recent studies (25, 27), a 10 mmHg difference in IASBPD was regarded as an upper limit of normal in our study. However, IASBPD ≥ 10 mmHg alone did not have a significant impact on CVD or all-cause mortality, it may be related to our small sample size. There were only 244 people in this group of IASBPD ≥ 10 mmHg without carotid plaque.

Our study found that carotid artery plaque and IASBPD synergistically increased the risks of CVD and all-cause mortality, especially the risk of cerebral ischemic stroke by 2.47 times. According to a recent cohort study of 117,407 people, IASBPD was associated with arterial stiffness, reflecting arteriosclerosis, but not with the presence of coronary artery calcium, reflecting atherosclerosis (28). Arteriosclerosis involves the structural changes of the arterial wall (the damage or degradation of the elastic fibers, or an increase in aggregating glycosaminoglycans or collagen fibers, or both), which affect the hemodynamic stress of the vascular wall leading to the increase of arterial stiffness finally (29). Atherosclerosis is a chronic process involving many factors, which starts from endothelial dysfunction, develops to intima thickening, and finally forms atherosclerotic plaque, characterized by lipid deposition in the inner walls of blood vessels and involving the inflammatory response (1). Therefore, arteriosclerosis and atherosclerosis are two pathological processes of vascular damage, which synergistically increase the risk of cardiovascular events. The combination of plaque and IASBPD may improve the identification of people at high risk for CVD. Based on the present, individuals with IASBPD ≥ 10 mmHg should be screened routinely for the presence of carotid artery plaque. In addition, some studies demonstrated that Lipid-lowering therapy helps stabilize, regress or even eliminate plaques (30, 31). Therefore, to reduce the burden of CVD and mortality, we suggest earlier early lipid-lowering therapy for the patients with IASBPD ≥ 10 mmHg and plaque together.

The strength of this study was the prospective large cohort for a long follow-up study. The combination of IASBPD and carotid plaque may become a simple, accessible, and non-invasive marker for screening individuals at high-cardiovascular risk, to guide patients to take intervention lifestyle or drug treatment as soon as possible. Besides, ascertainments of CVD and mortality were recorded based on the Municipal Social Insurance Institution and Hospital Discharge Register, which was updated annually during the follow-up period. As a result, we seldom lose the sample size to follow up.

However, some limitations still need to be considered. First, we only focused on whether the carotid artery plaque exists. Other features of the plaque including area, properties, and thickness were not measured, which may also be associated with CVD and mortality. The effect of carotid plaque characteristics on cardiovascular disease and mortality remains to be further studied. Second, we only measured IASBPD and plaque at baseline, but it represents the impact of vascular damage status on long-term prognosis. Third, our study included more men. Because, the study was based on the Kailuan study, from which the subjects were mostly male coal miner workers.

In conclusion, the combination of IASBPD and carotid artery plaque was associated with incident CVD and all-cause mortality. Therefore, early screening and intervention for IASBPD and carotid plaque may be a strategy to prevent CVD in the general population.

## Data availability statement

The data analyzed in this study is subject to the following licenses/restrictions: The datasets generated and/or analyzed during the current study are not publicly available, as per internal protocol, but are available from the corresponding author on reasonable request. Requests to access these datasets should be directed to HX.

## Ethics statement

The studies involving human participants were reviewed and approved by Ethics Committee of the Kailuan Medical Group, Kailuan Company. The patients/participants provided their written informed consent to participate in this study.

## Author contributions

HX and SW designed the research. SW and CY conducted the research. CY and QX wrote the manuscript. SC and JW analyzed the data. SZ, CW, MW, MZ, YS, and ZH edited the manuscript. All authors read and approved the final manuscript.

## Abbreviations

IASBPD: inter-arm systolic blood pressure difference
CVD: cardiovascular diseases
CI: cerebral infarction
MI: myocardial infarction
HRs: hazard ratios
CI: confidence interval
BP: blood pressure.

## Appendix

**Appendix 1:** The univariate analysis of the relevant risk factors.

		*P*	HR (95% CI)
CVD	Age	<0.01*	1.04 (1.03∼1.05)
	Gender	<0.01*	0.48 (0.36∼0.63)
	BMI	<0.01*	1.08 (1.04∼1.12)
	TC	>0.05	1.11 (0.99∼1.25)
	HDL	<0.01*	0.59 (0.43∼0.81)
	Smoking	<0.01*	1.85 (1.44∼2.37)
	Drinking alcohol	<0.01*	1.24 (1.06∼1.45)
	Hypertension	<0.01*	4.00 (3.00∼5.33)
	Diabetes	<0.01*	2.86 (2.15∼3.79)
	Taking hypotensive drugs	<0.01*	2.47 (1.91∼3.20)
All-cause mortality	Age	<0.01*	1.09 (1.08∼1.10)
	Gender	<0.01*	0.38 (0.28∼0.51)
	BMI	<0.05*	0.96 (0.92∼1.00)
	TC	>0.05	1.06 (0.95∼1.20)
	HDL	>0.05	0.98 (0.75∼1.29)
	Smoking	<0.01*	1.49 (1.17∼1.90)
	Drinking alcohol	>0.05	0.90 (0.76∼1.07)
	Hypertension	<0.01*	2.98 (2.29∼3.88)
	Diabetes	<0.01*	2.20 (1.65∼2.94)
	Taking hypotensive drugs	<0.01*	1.87 (1.31∼2.24)
CI	Age	<0.01*	1.05 (1.03∼1.06)
	Gender	<0.01*	0.48 (0.34∼0.66)
	BMI	<0.01*	1.08 (1.04∼1.04)
	TC	>0.05	1.04 (0.90∼1.20)
	HDL	<0.01*	0.48 (0.33∼0.69)
	Smoking	<0.01*	2.00 (1.49∼2.67)
	Drinking alcohol	<0.01*	1.28 (1.07∼1.54)
	Hypertension	<0.01*	3.09 (2.23∼4.27)
	Diabetes	<0.01*	4.12 (2.93∼5.81)
	Taking hypotensive drugs	<0.01*	2.95 (2.19∼3.97)
MI	Age	<0.01*	1.03 (1.01∼1.05)
	Gender	<0.01*	0.38 (0.20∼0.72)
	BMI	<0.05*	1.08 (1.01∼1.17)
	TC	<0.01*	1.40 (1.17∼1.68)
	HDL	>0.05	0.87 (0.47∼1.60)
	Smoking	<0.05*	1.85 (1.10∼3.12)
	Drinking alcohol	>0.05	1.20 (0.86∼1.67)
	Hypertension	<0.01*	3.10 (1.74∼5.54)
	Diabetes	>0.05	1.91 (0.99∼3.69)
	Taking hypotensive drugs	>0.05	1.46 (0.80∼2.67)

HR, hazard ratios; 95% CI, 95% confidence interval; CVD, cardiovascular disease; CI, cerebral ischemia; MI, myocardial infarction; BMI, body mass index; TC, total cholesterol; HDL, high density lipoprotein. *Statistically significant (*P* < 0.05).
